# Deep Learning-Based Classification and Targeted Gene Alteration Prediction from Pleural Effusion Cell Block Whole-Slide Images

**DOI:** 10.3390/cancers15030752

**Published:** 2023-01-25

**Authors:** Wenhao Ren, Yanli Zhu, Qian Wang, Haizhu Jin, Yiyi Guo, Dongmei Lin

**Affiliations:** Key Laboratory of Carcinogenesis and Translational Research (Ministry of Education), Department of Pathology, Peking University Cancer Hospital and Institute, Beijing 100142, China

**Keywords:** deep learning, pleural effusion, cell blocks, classification, gene alteration prediction

## Abstract

**Simple Summary:**

For many patients with advanced cancer, pleural effusion is the only accessible specimen for establishing a pathological diagnosis. Some pleural effusion cell blocks have not undergone adequate morphological, immunohistochemical, or genetic analysis due to problems with the specimen itself or cost. Deep learning is a potential way to solve the above problems. In this study, on the basis of scanning whole slide images of pleural effusion cell blocks, we investigated the identification of benign and malignant pleural effusion, the determination of the primary site of pleural effusion common metastatic carcinoma, and the alteration of common targeted genes using a weakly supervised deep learning model. We achieved good results in these tasks. Although deep learning cannot be the gold standard for diagnosis, it can be a useful tool to aid in cytology diagnosis.

**Abstract:**

Cytopathological examination is one of the main examinations for pleural effusion, and especially for many patients with advanced cancer, pleural effusion is the only accessible specimen for establishing a pathological diagnosis. The lack of cytopathologists and the high cost of gene detection present opportunities for the application of deep learning. In this retrospective analysis, data representing 1321 consecutive cases of pleural effusion were collected. We trained and evaluated our deep learning model based on several tasks, including the diagnosis of benign and malignant pleural effusion, the identification of the primary location of common metastatic cancer from pleural effusion, and the prediction of genetic alterations associated with targeted therapy. We achieved good results in identifying benign and malignant pleural effusions (0.932 AUC (area under the ROC curve)) and the primary location of common metastatic cancer (0.910 AUC). In addition, we analyzed ten genes related to targeted therapy in specimens and used them to train the model regarding four alteration statuses, which also yielded reasonable results (0.869 AUC for ALK fusion, 0.804 AUC for KRAS mutation, 0.644 AUC for EGFR mutation and 0.774 AUC for NONE alteration). Our research shows the feasibility and benefits of deep learning to assist in cytopathological diagnosis in clinical settings.

## 1. Introduction

Serous effusion cytology is a common clinical method used to differentiate benign from malignant serous effusions due to its minimal discomfort and risk to patients [1,2,3]. With the gradual increase in treatment methods and the emergence of cell block technology, clinical cytologists are required not only to distinguish benign and malignant pleural effusions but also to identify the primary location of metastatic carcinomas or mutant genes using auxiliary methods such as immunohistochemistry or molecular detection [4,5]. However, due to the subjective and regionally dependent diagnostic level of cytopathologists, there is a problem of low consistency in the diagnosis of benign and malignant pleural effusions [3,6]. In addition, malignant pleural effusions have not yet been recognized as routine substrates for the immunohistochemical or molecular testing pipeline due to their occasionally low tumor fraction and sparse cellularity [7,8,9]. Low tumor cellularity means it is not always possible to perform sufficient immunohistochemical and molecular analyses to accurately diagnose gene mutation and the primary site of metastatic cancer. In cases with sufficient cellularity, the cost burden is another reason some patients fail to undergo immunohistochemistry or genetic testing.

In recent years, artificial intelligence in the form of deep learning has been extensively utilized in the field of pathology and has the potential to solve several clinical pathology problems [10,11,12,13], but fewer studies have focused on clinical cytopathology [14,15]. In this study, we used a weakly supervised deep learning approach to investigate the determination of benign and malignant pleural effusion, the identification of the primary site of metastatic cancer, and the prediction of genetic alterations associated with targeted therapy using whole-slide images (WSIs) of pleural effusion cell blocks in an effort to solve some urgent clinical issues with deep learning.

## 2. Materials and Methods

### 2.1. Materials

From January 2018 to September 2022, 1321 consecutive pleural effusion specimens from Peking University Cancer Hospital were embedded to the greatest extent possible, and the successfully embedded cases were then scanned as whole-slide images (WSIs) with Pannoramic 250 Flash III scanner (3DHISTECH, Hungary). Several WSIs with unclear scanning were rescanned. Patient demographics, clinical presentation, cytology and histology reports, auxiliary tests, and patient management information were extracted from pathology databases and electronic medical records. All sections were assessed blindly by two senior cytopathologists (W.R. and Y.Z.), and in the case of inconsistent diagnosis, a unified diagnosis was negotiated with the participation of a third cytopathologist (Q.W.). Ultimately, 1307 digitized WSIs were included in the subsequent analysis.

For all malignant tumors, cases with a clear and unique tumor history did not confirm their primary site through immunohistochemistry, whereas the remaining cases (those with multiple prior malignancies or ambiguous primary locations) were confirmed with immunohistochemistry.

The AmoyDx^®^ Essential next-generation sequencing (NGS) Panel (Amoy Diagnostics, Xiamen, China) was used to detect genetic abnormalities in FFPE cell block tumor tissues (http://www.amoydiagnostics.com/productDetail_9.html, accessed on 1 January 2023). The AmoyDx^®^ Essential NGS Panel is an NGS-based in vitro diagnostic assay intended for qualitative detection of single nucleotide variants (SNVs), insertions and deletions (InDels), gene fusions, and copy number variations (CNVs) in driver genes. An amplification refractory mutation system polymerase chain reaction (ARMS-PCR) and a mutation detection kit (Amoy Diagnostics) were used to identify the gene alteration in driver genes. This kit is designed for the detection of common mutations in 10 genes in two categories: (1) mutation gene detection (EGFR gene (exons 18, 19, 20, 21), BRAF gene (V600E mutation), KRAS gene (codons 12 and 13 of exon 2), NRAS gene (codon 61 of exon 3), HER2 gene (exon 20), PIK3CA gene (exons 9 and 20), MET gene (exon 14 skipping mutation)); and (2) fusion gene detection (ALK, ROS1, RET fusion gene detection). In this study, we labeled a gene’s alteration status as “NONE” if none of the 10 genes listed above contained abnormalities.

### 2.2. Datasets

All the HE-stained slides were digitized with a 40× magnification objective and a resolution of 0.25 µm/pixel and saved in MRXS format according to the manufacturer’s protocol (Pannoramic 250 Flash III scanner, 3DHISTECH, Budapest, Hungary).

**Figure 1:**
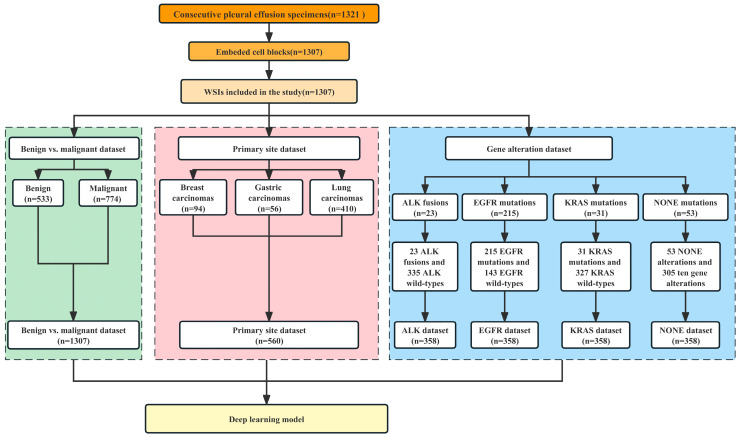
Case screening and establishment of datasets for three tasks.

The dataset was partitioned into three parts as shown in Figure 1: (1) the benign vs. malignant dataset which was used to differentiate between benign and malignant pleural effusion and contained 1307 WSIs (representing 533 benign lesions and 774 malignant tumors); (2) the primary site dataset which was used to identify the primary site of common metastatic cancers and contained 560 WSIs (representing 94 breast invasive ductal carcinomas, 56 gastric adenocarcinomas, and 410 lung adenocarcinomas); and (3) the gene alteration dataset, which was further divided into the ALK dataset (23 ALK fusions and 335 ALK wild-types), the EGFR dataset (215 EGFR mutations and 143 EGFR wild-types), the KRAS dataset (31 KRAS mutations and 327 KRAS wild-types) and the NONE dataset (53 NONE alterations and 305 gene mutations).

According to the distribution of the dataset, whole-slide image (WSI) cases were randomly assigned to the training set, validation set, and test set in a ratio of 6:2:2. In order to solve the problem of unbalanced data, we performed different levels of data augmentation (such as rotating, random flipping, Gaussian blurring, and clipping) on the training set in different datasets. To analyze the predictive performance of the model more accurately, we employed the 10-fold cross-validation method.

### 2.3. Image Preparation

#### 2.3.1. Preprocessing

**Figure 2:**
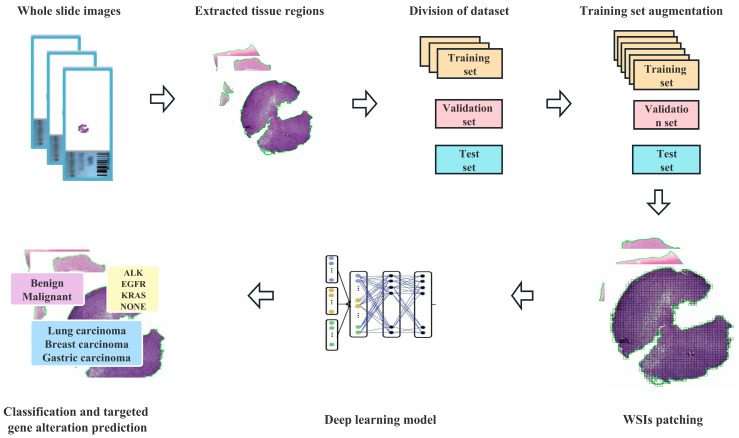
Flowchart of the deep learning framework presented in this study.

Our pipeline (as shown in Figure 2) began with automatic segmentation of the tissue regions based on the operations associated with the Python Open-CV application programming interface. Except for the manual markings that we removed from the slides before scanning, we did not undertake any extra work, such as stain normalization or removing artifacts, on our images and used the entire tissue region of each slide during evaluation. Because the sizes of the tissue regions were still too large (~1.62 GB per image) for direct input into a neural network, all the tissue regions were cropped from the original microscopic images without overlapping and then resized to 299 × 299 pixels as input for model training with Qupath (Version 0.3.0) [16]. The number of patches per slide depended on the specimen size, and mean slide patches were 7989 ± 4568.

#### 2.3.2. Deep Learning Model Training

In our approach, inception-ResNet-v2 serves as the backbone [17], and the weakly supervised WSI classification model is based on multiple instance learning (MIL), which treats each WSI as a collection of many smaller regions or patches. According to MIL, a slide should be labeled as positive if at least one of its patches is in the positive class, and as negative if all of its patches are in the negative class [18]. In order to improve the interpretability of the model, we added an attention-based pooling function to the model [19]. Using attention-based learning, our model can generate interpretable heatmaps that enable clinicians to visualize the regions that the model focuses on when making a prediction. Without pixel-level annotation during training, we can determine for every tissue region the relative contribution to and correlation with the model prediction.

During training, the slides were randomly sampled using a batch size of 299. The pretrained classifier inferred all of the patches in the training dataset using the weights learned from the ImageNet dataset [20]. The attention module’s weights and bias parameters were initialized at random, and the module was trained in conjunction with the rest of the model using the slide-level labels. To generate the slide-level prediction for inference during validation and testing, we utilized the model to create predictions for each patch on the slide and then averaged their probability scores, according to the method of a previous study [21]. 

If the validation loss did not reduce during a span of 20 validation epochs, the model was terminated early. This process was repeated every 100,000 patches. The model with the lowest validation loss at each checkpoint was chosen for evaluation on the test set. We used a cross-entropy loss function and optimized the models’ parameters with stochastic gradient descent and the Adam optimizer at a learning rate of 0.0002 and a weight decay of 0.00001. To illustrate the relative contribution and significance of each tissue location, heatmaps were utilized.

### 2.4. Hardware and Software for Data Processing

We used multiple hard drives to store the raw files of the WSIs. Segmentation and patching of WSIs were performed on a computer with 40 Intel(R) Xeon(R) CPU E5-2640 v4 @ 2.40 GHz and Qupath (Version 0.3.0). Model training, validation, and testing were accelerated through data batch parallelization across three NVIDIA A5000 on local workstations. Our whole-slide processing pipeline is written in Python (3.6.2) and utilizes image-processing packages such as OpenSlide (3.4.1), OpenCV (4.1.1), and NumPy (version 1.18.1). The TensorFlow (version 2.9.0) deep learning library was used to load data and train deep learning models.

### 2.5. Evaluation Metrics and Graph

We measured model performance using AUC (area under the ROC curve), accuracy, sensitivity, specificity, positive predictive value (PPV), negative predictive value (NPV), and F1-score at the slide level. All plots were generated in R version 4.1.0.

## 3. Results

### 3.1. Patient Demographics and Clinicopathological Characteristics

**Figure 3:**
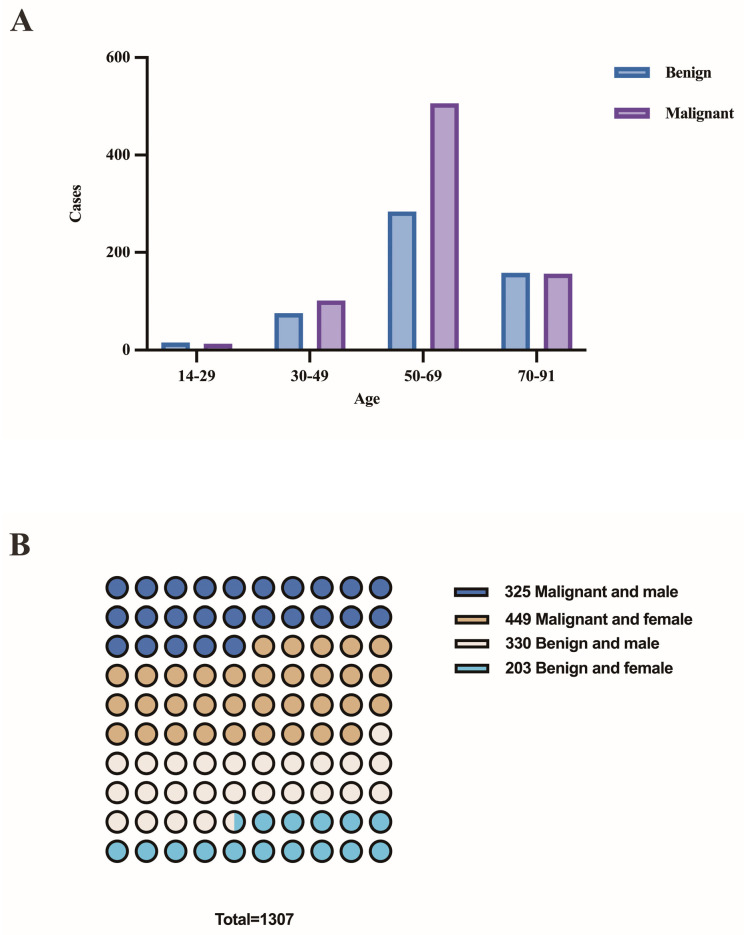
(**A**) The distribution of benign and malignant pleural effusions by age. (**B**) The distribution of benign and malignant pleural effusions by sex.

**Figure 4:**
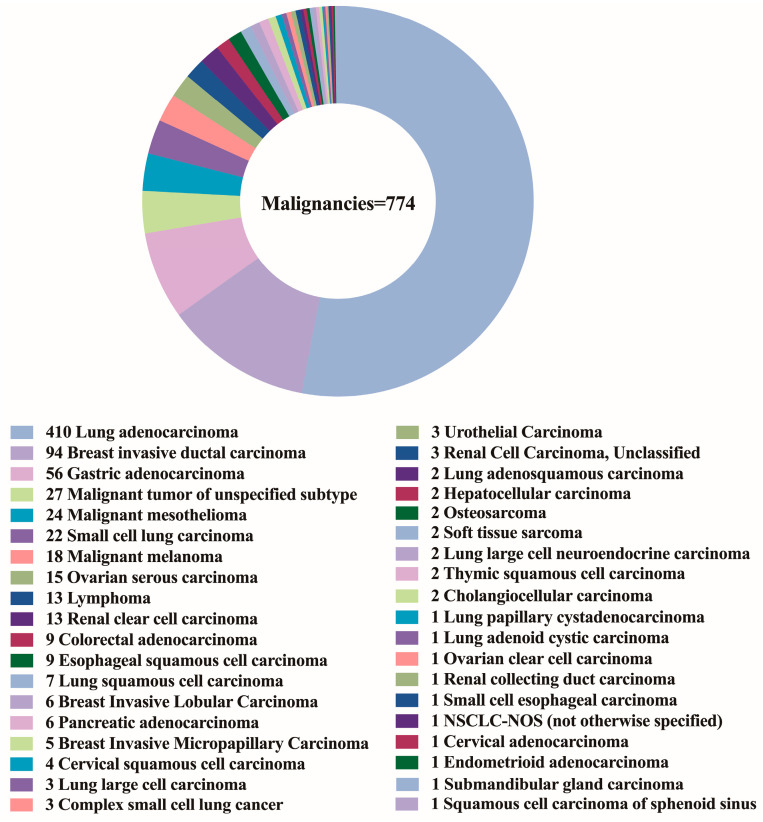
The specific pathological categories of 774 cases of malignant pleural effusion.

**Figure 5:**
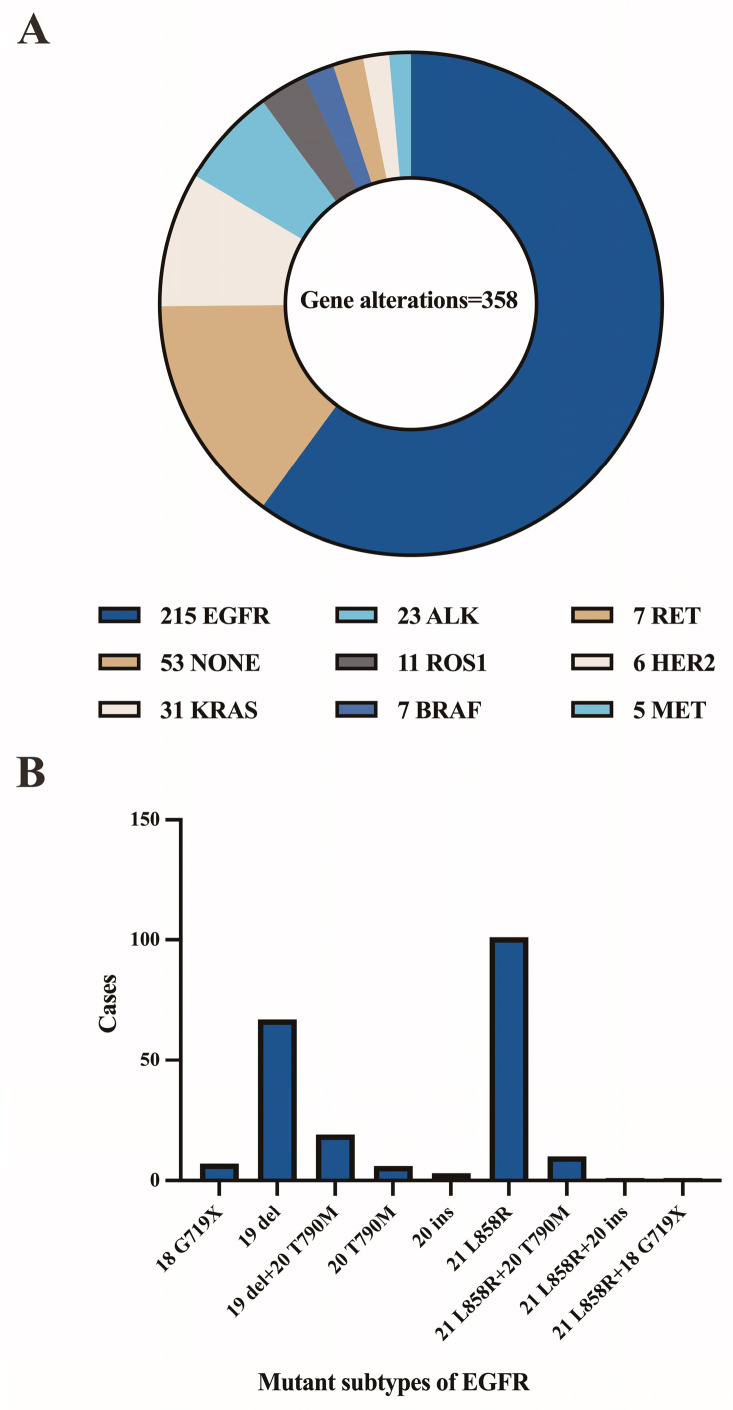
(**A**) The specific types of gene alterations. (**B**) Number of mutant subtypes of EGFR.

The patient population consisted of 655 males and 652 females, with a median age of 62 years (range 14–91 years). Figure 3A,B depicts the distribution of benign and malignant pleural effusions by age and sex. One hundred and eight instances (14.0%) among 774 malignancies had pleural effusion as the initial symptom, while 10 cases (1.3%) had multiple prior tumors. Malignant WSI that contained fewer than 10 tumor cells (contained very few tumor cells) were present in 7.0% (54/774), 10–100 tumor cells were present in 29.8% (231/774), and more than 100 tumor cells were present in 63.2% (489/774). Figure 4 depicts the specific pathological categories of 774 cases of malignant pleural effusion, the most prevalent of which were lung adenocarcinomas (410, 53.0%), breast invasive ductal carcinomas (94, 12.1%), and gastric adenocarcinomas (56, 7.2%). In 360 of 410 cases of metastatic lung cancers, genetic testing was successfully performed. Two cases containing two types of genetic mutations were excluded from subsequent analysis, and a total of 358 cases with a single gene alteration were included in subsequent predictive analysis of genetic alterations. The specific types of gene alterations are shown in Figure 5A,B. The most prevalent mutations were EGFR mutations (215/358, 60.1%), ALK fusions (23/358, 6.4%), KRAS mutations (31/358, 8.7%), and NONE alterations (53/358, 14.8%). 21 L858R (101/215, 47.0%) and 19 del (67/215, 31.2%) were the most common EGFR mutant subtypes.

### 3.2. Deep Learning Models for Differentiating between Benign and Malignant Pleural Effusions

**Figure 6:**
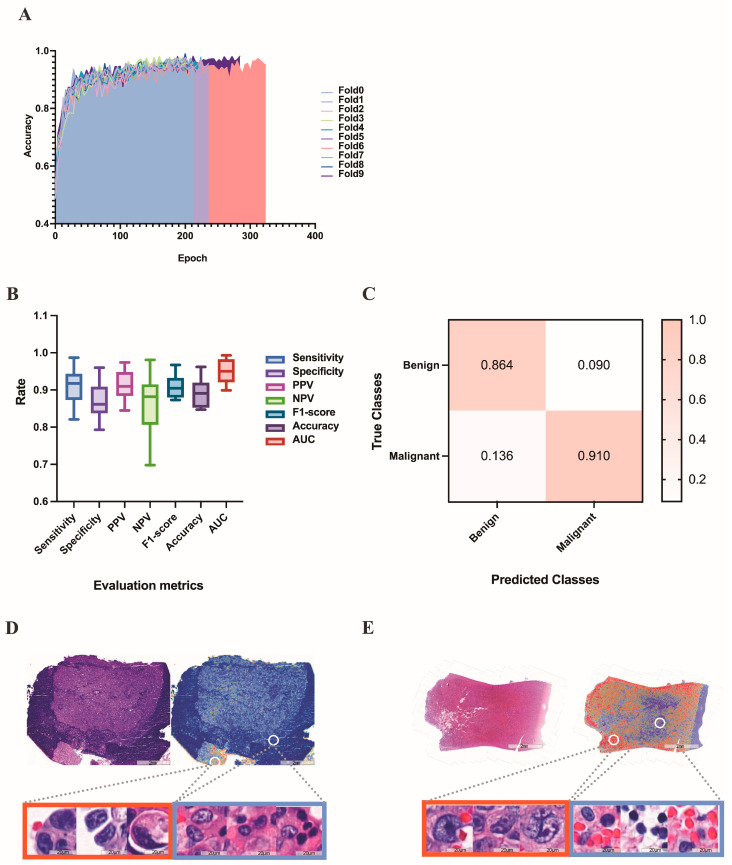
Results of the deep learning model for differentiating benign and malignant pleural effusion. (**A**) The learning curve of the deep learning model in distinguishing between benign and malignant WSI images. (**B**) Evaluation metrics in test set of benign vs. malignant dataset. (**C**) Confusion matrix in test set of benign vs. malignant dataset. (**D**) A HE-stained image of breast invasive ductal carcinoma, corresponding heatmap and magnified pictures of different attention regions in the heatmap. The redder the color, the higher the confidence of the malignancy. (**E**) Another example of an HE-stained image of gastric adenocarcinoma, corresponding heatmap and magnified pictures of different attention regions in the heatmap.

According to our research aim, we initially trained our model to distinguish between “benign” and “malignant” WSIs, with the terms simply representing the pathological characterization of the sampled cells. This task was trained on 320 benign and 464 malignant WSIs, validated on 107 benign and 155 malignant WSIs, and tested on 106 benign and 155 malignant WSIs. In terms of data set division, the benign and malignant tumors were randomly allocated to the training set, validation set, and test set in proportion as a whole. We did not deliberately allocate equal proportions to different types of malignant tumors. Since we adopted the 10-fold cross-validation method, the bias caused by the imbalance in the proportions of different types of malignant tumors in the training set, verification set, and test set can be reduced to a certain extent. The learning curve, evaluation metrics and confusion matrix of this task are shown in Figure 6A–C. In the test set, the results yielded an average AUC of 0.932 (range: 0.899 to 0.993), an average accuracy of 0.891 (range: 0.847 to 0.962), an average sensitivity of 0.911 (range: 0.821 to 0.987), an average specificity of 0.870 (range: 0.793 to 0.960), an average PPV of 0.910 (range: 0.845 to 0.974), an average NPV of 0.864 (range: 0.698 to 0.981), and an average F1-score of 0.909 (range: 0.873 to 0.967). The heatmaps (as shown in Figure 6D-E) demonstrate that our models are generally capable of identifying the boundary between malignant and benign tissue and are able to differentiate between tumor and nearby normal tissue without the usage of normal slides or region-of-interests during training. The largest proportion of false-negative cases was WSI with very few tumor cells (49.3%), and the largest proportion of false-positive cases was WSI with hyperplastic mesothelial cells or hyperplastic lymphocytes (48.6%).

### 3.3. Deep Learning Models in the Identification of the Primary Site of Metastatic Cancer

**Figure 7:**
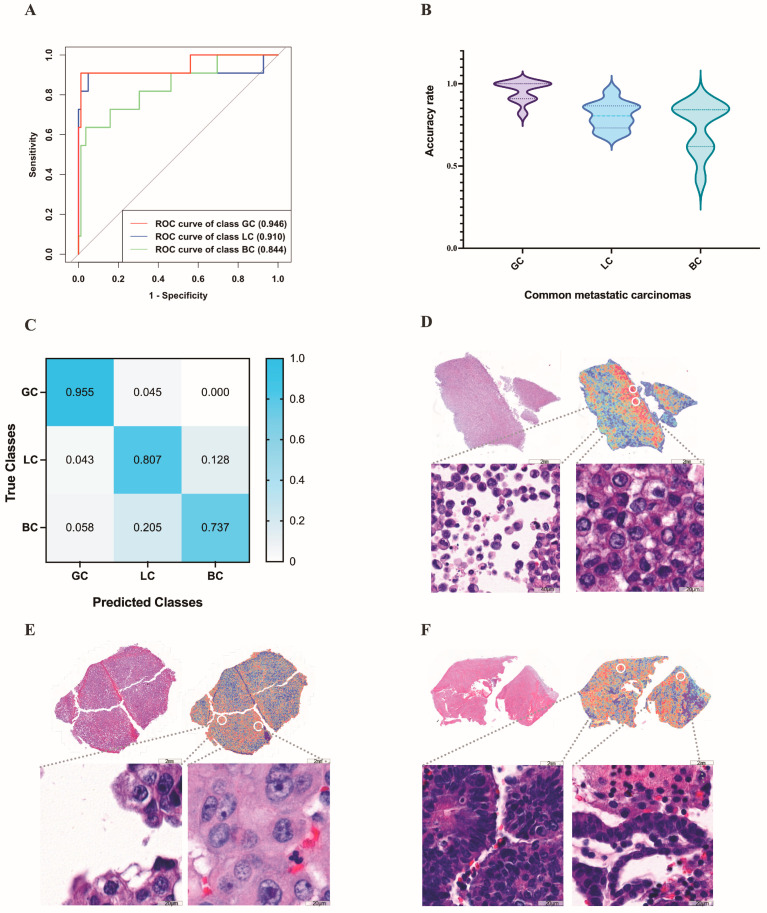
Results of the deep learning models in the identification of the primary site of metastatic carcinoma. (**A**) The ROC curve used for the common metastatic carcinomas on the test set. (**B**) The average accuracy rate of common metastatic carcinomas predicted by deep learning. (**C**) The confusion matrix used for the common metastatic carcinomas on the test set. (**D**–**F**) High-attention regions in the classification tasks. The gastric adenocarcinoma (**D**) highlights predominantly scattered isolated malignant cells, whereas the trained model for lung adenocarcinoma (**E**) emphasizes clusters with unregular borders and cellular pleomorphism. For breast invasive ductal carcinoma (**F**), the model emphasizes acini/glands or round cell groups. GC: gastric adenocarcinoma; LC: lung adenocarcinoma; BC: breast invasive ductal carcinoma.

Next, we evaluated the performance of our method on the more difficult task of differentiating between pleural effusions of common metastatic carcinomas. The task was trained on 224 cases of breast carcinoma (augmented by 56 cases of breast carcinoma), 238 cases of gastric adenocarcinoma (augmented by 34 cases of gastric adenocarcinoma), and 246 cases of lung adenocarcinoma. It was validated on 19 cases of breast carcinoma, 11 cases of gastric adenocarcinoma, and 82 cases of lung adenocarcinoma, and tested on 19 cases of breast carcinoma, 11 cases of gastric adenocarcinoma, and 82 cases of lung adenocarcinoma. Because the conventional MIL method, which was intended and widely implemented for weakly supervised positive/negative binary classification (for example, cancer versus normal), was not suitable for this three-category task, we performed learning with the mMIL method [22], which shows a good classification effect in multiclassification tasks. In the test set, this process resulted in an average AUC of 0.910 (range: 0.879 to 0.960) and an average accuracy of 0.810 (range: 0.750 to 0.884), as shown in Figure 7A. Among these, the average accuracy rates were 0.955 for gastric adenocarcinoma, 0.737 for breast invasive ductal carcinoma, and 0.807 for lung adenocarcinoma (Figure 7B). Figure 7C shown the confusion matrix of this task.

It is also important that in this classification task, the high attention regions of the deep learning model were consistent with the areas that cytopathologists focus on when making a diagnosis. For example, the trained model for gastric adenocarcinoma highlights predominantly scattered isolated malignant cells (Figure 7D) and uses them as strong evidence (high attention) for gastric adenocarcinoma, whereas the trained model for breast invasive ductal carcinoma emphasizes acini/glands or round cell groups (Figure 7E). For lung adenocarcinoma, the model emphasizes clusters with unregular borders and cellular pleomorphism (Figure 7F), which are consistent with human pathology expertise. 

Misclassified gastric adenocarcinomas were all predicted to be lung adenocarcinomas. For misclassified breast cancers, 78% of them were predicted to be lung adenocarcinomas and 22% were predicted to be gastric adenocarcinomas. Of the misclassified lung adenocarcinomas, 33.5% were predicted to be gastric adenocarcinomas and 66.5% were predicted to be breast cancers.

### 3.4. Predicting Gene Alteration Status from Whole-Slide Images Using Deep Learning Models

**Figure 8:**
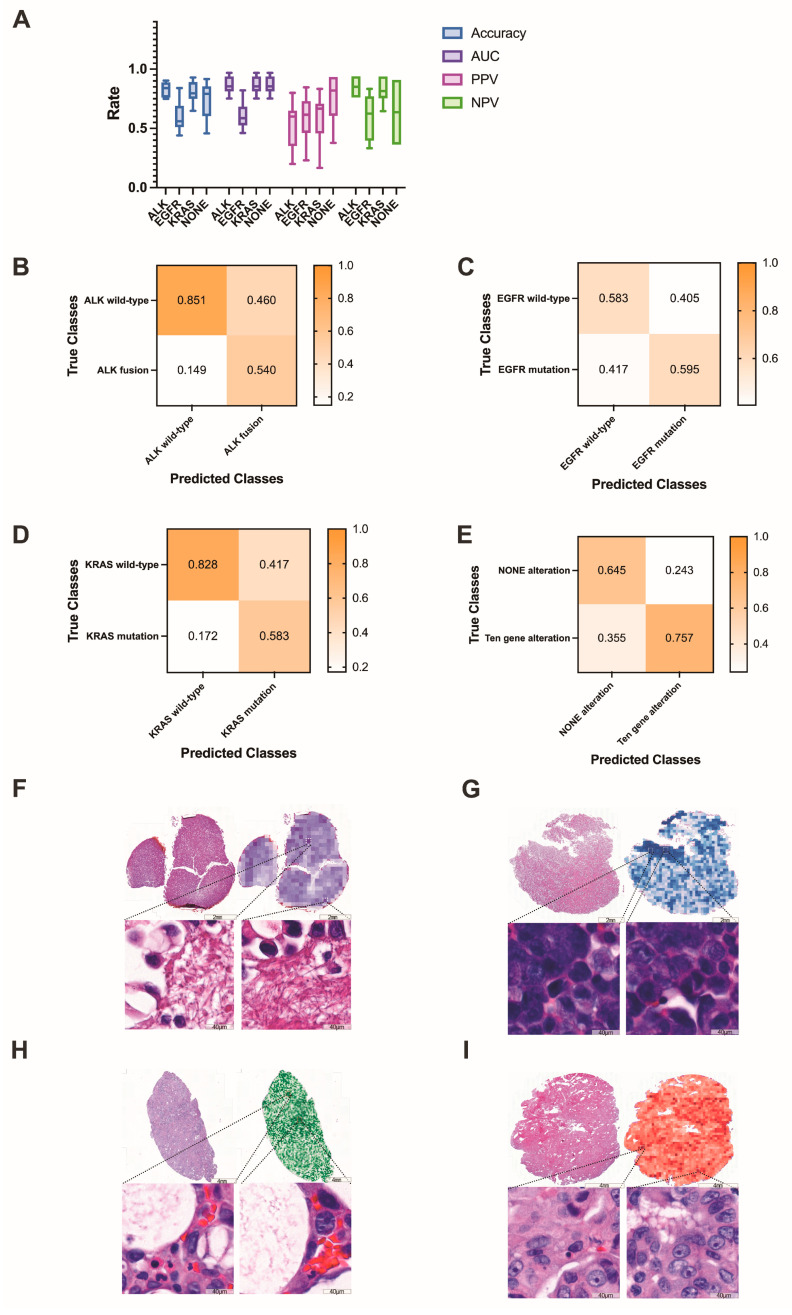
(**A**) The prediction results of common gene alterations in lung adenocarcinoma. (**B**–**E**) The confusion matrices of common gene alteration. (**F**–**I**) Examples of the heatmaps of ALK fusion, EGFR mutation, KRAS mutation and NONE alteration, respectively. The darker the color, the higher the confidence of the corresponding gene alteration.

Next, we focused on lung adenocarcinoma WSIs and examined whether deep learning can be trained to predict gene alterations using only images as the input. To ensure that the training and test sets comprised sufficient images of gene alteration cases, we only selected common gene alterations as the target of this classification task. We investigated each of the common genes individually using binary classification. The prediction results for each common gene are shown in Figure 8A. The confusion matrices of common gene alteration are shown in Figure 8B–E. The heatmaps of common gene alteration are shown in Figure 8F–I.

In the ALK dataset, the training set contained 345 ALK fusion (augmented by 23 ALK fusion) and 335 ALK wild-type WSIs, the validation set contained 13 ALK fusion and 201 ALK wild-type WSIs, and the test set contained 5 ALK fusion and 67 ALK wild-type WSIs. The results yielded an average AUC of 0.869 (range: 0.752 to 0.969), accuracy of 0.829 (0.750 to 0.903), PPV of 0.540 (0.200 to 0.800), and NPV of 0.850 (0.761 to 0.940) in the test set.

The KRAS dataset consisted of 31 KRAS mutations and 327 KRAS wild-type WSIs. After training on 190 KRAS mutations (augmented by 19 KRAS mutations) and 197 KRAS wild-type WSIs, the test set (6 KRAS mutation and 65 KRAS wild-type) yielded an average AUC of 0.804 (0.635 to 0.977), accuracy of 0.807 (0.648 to 0.930), PPV of 0.583 (0.166 to 0.833), and NPV of 0.828 (0.646 to 0.938).

For EGFR mutation or wild-type, the test set contained 43 EGFR mutations and 29 EGFR wild-type WSIs. The average AUC, accuracy, PPV, and NPV were 0.644 (0.468 to 0.821), 0.592 (0.480 to 0.840), 0.600 (0.231 to 0.846), and 0.583 (0.333 to 0.833), respectively. For ten gene alterations and NONE alterations, the test set contained 11 NONE-alteration and 61 gene-alteration WSIs. The results yielded average AUC, accuracy, PPV, and NPV values of 0.774 (0.615 to 0.879), 0.740 (0.458 to 0.917), 0.757 (0.377 to 0.934) and 0.645 (0.364 to 0.909), respectively.

## 4. Discussion

To the best of our knowledge, this is the largest study to date evaluating the application of deep learning to the cytological diagnosis of pleural effusion cell blocks. Deep learning focused in the direction of cytopathology is less available and has not been applied in more complex clinical scenarios. The majority of studies have only performed the differentiation of benign and malignant pleural fluid [23,24,25], and some research has studied only the most frequently mutated genes in lung adenocarcinoma [21], although these genes have no guiding meaning in target therapy. In addition, there are few publications on the use of deep learning to forecast the primary site of metastatic tumors. Identifying the primary site of metastatic cancer is a critical diagnostic task. Many patients present with pleural effusion as their first symptom, and different primary sites can lead to very different treatments [26].

In this study, we evaluated the use of deep learning to distinguish benign pleural effusion from malignant pleural effusion. Due to cytomorphologic overlap, proliferating mesothelial cells are frequently difficult to distinguish from cancer cells in routine cytopathological diagnostic procedures [27]. Our study demonstrates that a deep learning model can be used to aid in diagnostic work; it can classify normal and malignant effusions with an AUC of 0.932, an accuracy of 0.891, a sensitivity of 0.911, a specificity of 0.870, a PPV of 0.910, an NPV of 0.864, and an F1-score of 0.909. In institutions where there is a low level of diagnostic expertise, there is benefit to be gained from the use of deep learning systems. We further analyzed the cases in which the deep learning model misjudged, and found that the main reason for the false negative may be that the WSI contains very few tumor cells (accounting for 59.3%), and the deep learning model fails to make correct judgments based on these few tumor cells. The main causes of false positives are proliferating lymph node cells and hyperplastic mesothelial cells (48.6%), proliferating lymphocytes may not be well distinguished from lymphoma, small cell carcinoma, etc., and hyperplastic mesothelial cells may be indistinguishable from some malignant tumors with similar morphology, such as mesothelioma, gastric cancer, etc., resulting in incorrect prediction by deep learning systems.

Second, we examined the applicability of our model to the determination of the primary site of metastatic cancer in pleural fluid. Significant cytomorphologic overlap exists between carcinomas of different primary origins, and immunohistochemistry is frequently needed to determine the primary site. However, some pleural effusions of unknown primary may not be confirmed by limited immunohistochemical items due to the low cellularity in the cell block [28,29]. In our study, after training and testing on 560 WSIs (94 breast invasive ductal carcinomas, 56 gastric adenocarcinomas, and 410 lung adenocarcinomas), we discovered that our deep learning model can distinguish between the three types of metastatic cancer (0.910 AUC), which is of great benefit to patients with only pleural effusion specimens available when the cell block contains sparse tumor cells. According to the predictions of deep learning, the primary site is likely to be verified by only two or three immunohistochemistry markers; deep learning can considerably improve the confirmation rate of the primary site. It is worth mentioning that in clinical work, the prediction of rare tumors is more clinically significant, because the primary lesions of rare tumors often require a larger immunohistochemical panel to be clear, and the prediction of rare tumors by deep learning prediction systems can reduce the workload and patient costs. In our study, we did not make predictions for rare tumors due to the small number of rare tumors and the lack of public datasets available for pleural effusion cell blocks, but we shared our WSIs data in the hope that future studies can integrate our data to complete deep learning predictions for rare metastatic cancer in pleural fluid.

When analyzing the misclassified cases, gastric adenocarcinoma achieved a good accuracy, which may be caused by the relatively single morphology of metastatic gastric adenocarcinoma. In our study, metastatic gastric adenocarcinoma cells in pleural fluid were all scattered isolated malignant cells, and only 0.045% of gastric adenocarcinoma was misclassified as lung adenocarcinoma. The main cause of breast carcinoma misclassification (60%) is that some poorly differentiated breast invasive ductal carcinomas exhibiting as three-dimensional round cell groups were misclassified as solid lung adenocarcinoma. Lung adenocarcinoma has a variety of morphologies, including three-dimensional groups in papillary configurations, proliferation spheres or single-cell scattered forms, or a combination of multiple forms, and the varied morphology may cause its morphological overlap with breast and gastric adenocarcinoma, thereby reducing the accuracy of predictions.

We studied the feasibility of predicting targeted mutations using WSI images of pleural effusion cell blocks. Numerous clinical investigations have demonstrated that gene alteration status is a major predictor of the success of targeted therapy. The presence of ALK and ROS1 gene fusions correlates with the efficacy of ALK/MET inhibitor therapy. Patients with RET fusion could benefit from MET/RET/VEGFR inhibitors. BRAF-mutated patients benefit from BRAF inhibitor therapy, and KRAS/NRAS/HER2/PIK3CA mutation status is associated with the prognosis of some targeted drugs [30,31]. According to the National Comprehensive Cancer Network Guidelines for non-small cell lung cancer, gene mutation testing is essential prior to targeted therapy, and multitarget testing is strongly suggested for the most effective precision oncology treatment. Given the significance and impact of these genetic abnormalities, the ability to anticipate genetic alterations from pathology images rapidly and affordably may aid in the treatment of cancer patients. However, pleural effusion samples are not usually sufficient for a comprehensive analysis of targeted mutations [32]. Although some gene mutations have certain morphological characteristics [33], the positive rate of gene mutation prediction based on morphology is still low. To improve the success rate of gene detection, we used deep learning to predict the gene of most likely change.

In general, the results of our model in predicting targeted mutations are reasonable. Our model acquired good performance in predicting ALK fusion and KRAS mutation (0.869 AUC and 0.804 AUC), respectively, whereas the performance was relatively poor in predicting EGFR and NONE alterations. We suggest that the reason for the poor prediction accuracy of EGFR and NONE alterations is that they have more mutant subtypes. In our study, the mutant subtypes of EGFR are depicted in Figure 5B. Different mutant subtypes may result in different morphological alterations, thereby decreasing prediction accuracy. In addition, only ten genes were identified in our study, and cases with NONE alterations may contain altered genes other than the ten genes. The genetic complexity may further lead to the confounding of the corresponding morphology, consequently diminishing the accuracy of the prediction. In addition, the small amount of training data is a limiting factor for achieving higher accuracy. Therefore, we have shared the WSIs and corresponding genetic alteration information from this study so that future investigations based on using genetic subtypes and increasing the number of training instances may be able to further improve the prediction accuracy.

Moreover, we found that the attention heatmaps exhibited a high level of agreement with the cytologist’s target region when tested on the benign vs. malignant dataset and primary site dataset, which gives us great hope and demonstrates the interpretability and dependability of our model. Although the accuracy of our model in predicting gene alterations is not very high, it is possible to detect morphological features associated with gene abnormalities if future research improves prediction accuracy.

Our research has the following limitations: (1) This was a single center, data-based study. There is no publicly available dataset for pleural effusion WSI images. Although we took clinically consecutive instances and did not normalize our WSI images to improve the robustness of the model, our model needs to be verified on a multicenter and larger dataset. In an effort to address the paucity of data on pleural effusion, we have made the data from this study available to researchers interested in additional investigation. (2) Due to the small number of cases with uncommon metastatic cancer and unusual mutations, our analysis was unable to forecast these conditions. More rare cases should be included in future studies to develop a more clinically applicable deep learning model.

## 5. Conclusions

Overall, this study implies that a deep learning model based on the pleural effusion cell blocks may be an effective diagnostic tool for cytopathologists. In addition to being able to differentiate between benign and malignant pleural effusion, the model can also identify the primary site of common metastatic malignancies, allowing for more precise medical treatment of patients. Precision medicine focuses increasingly on the genetic alterations of the disease, and different mutations result in different targeted therapies. Our model performs well in predicting KRAS mutations and ALK fusions, but further improvements are needed in predicting EGFR mutations and NONE mutations. Future research could analyze the subtypes of targeted mutations and collect more rare pleural effusion metastatic carcinomas and rare mutations to improve the model’s accuracy. Although deep learning cannot be the gold standard for diagnosis, it can be a useful tool to aid in cytology diagnosis. Immunohistochemistry and genetic tests can achieve their goals more efficiently with the guidance of deep learning.

## Data Availability

The datasets generated and/or analyzed during the current study are available online at https://www.aliyundrive.com/s/cUnri7B82Qp (accessed on 1 January 2023).
